# The Making and Breaking of Serine-ADP-Ribosylation in the DNA Damage Response

**DOI:** 10.3389/fcell.2021.745922

**Published:** 2021-11-15

**Authors:** Kira Schützenhofer, Johannes Gregor Matthias Rack, Ivan Ahel

**Affiliations:** Sir William Dunn School of Pathology, University of Oxford, Oxford, United Kingdom

**Keywords:** DNA damage, PARP, ADP-ribosylation, cancer, PARG, neurodegeneration, posttranslational modification (PTM), ARH3

## Abstract

ADP-ribosylation is a widespread posttranslational modification that is of particular therapeutic relevance due to its involvement in DNA repair. In response to DNA damage, PARP1 and 2 are the main enzymes that catalyze ADP-ribosylation at damage sites. Recently, serine was identified as the primary amino acid acceptor of the ADP-ribosyl moiety following DNA damage and appears to act as seed for chain elongation in this context. Serine-ADP-ribosylation strictly depends on HPF1, an auxiliary factor of PARP1/2, which facilitates this modification by completing the PARP1/2 active site. The signal is terminated by initial poly(ADP-ribose) chain degradation, primarily carried out by PARG, while another enzyme, (ADP-ribosyl)hydrolase 3 (ARH3), specifically cleaves the terminal seryl-ADP-ribosyl bond, thus completing the chain degradation initiated by PARG. This review summarizes recent findings in the field of serine-ADP-ribosylation, its mechanisms, possible functions and potential for therapeutic targeting through HPF1 and ARH3 inhibition.

## Introduction

**FIGURE 1:**
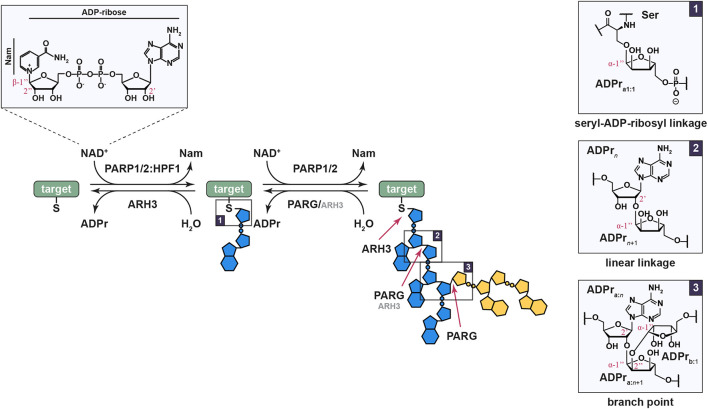
Ser-ADPr is a reversible and complex modification. The reaction involves the transfer of the ADPr moiety from β-NAD^+^ under inversion at the anomeric carbon, thus resulting in a modification in the α-conformation. The initial modification of a serine residue is catalyzed by the PARP1/2:HPF1 complex (box 1), while further chain extension is catalyzed by PARP1/2 alone. The latter occurs as linear, ribose(1″→2′)ribose (box 2), or infrequently branched, ribose(1″→2″)ribose(1″→2′)ribose (box 3), continuations leading to a large and diverse polymer structure. Linear polymers are primarily degraded by PARG, and to a lesser extend ARH3, while branch pruning, hydrolysis of the 1″→2″ bond, is carried out solely by PARG and precedes the cleavage of the 1″→2′ bond at branch points. In contrast, the proximal seryl-ADP-ribosyl bond can only be cleaved by ARH3. Identified target proteins in the context of the DDR include PARP1 and 2 themselves (automodification), histones (primarily H2B, H3, H4, and H1), FEN1, LIG3, and NUCKS1.

ADP-ribosylation refers to the transfer of ADP-ribose (ADPr) moiety from NAD^+^ onto substrate proteins or nucleic acids by enzymes termed (ADP-ribosyl)transferases (ARTs; Figure 1; Liu and Yu, 2015; Wei and Yu, 2016; Munnur and Ahel, 2017; Zarkovic et al., 2018; Munnur et al., 2019; Groslambert et al., 2021). ADP-ribosylation can occur as mono- or poly(ADP-ribosyl)ation (MARylation or PARylation, respectively) and is a highly conserved and widespread posttranslational modification (PTM) that controls many cellular processes, including cell proliferation and differentiation, the cellular stress response, maintenance of genome stability, behavior, viral infection, and microbial metabolism (Perina et al., 2014; Wei and Yu, 2016; Cohen and Chang, 2018; Palazzo et al., 2019; Crawford et al., 2021; Mikolčević et al., 2021). Proteins participating in ADPr signaling are often described in terms of “writers,” i.e., ARTs, “readers” that contain ADPr-binding domains, and “erasers” which modify or remove the ADP-ribosylation signal (Gupte et al., 2017).

One of the ART families, the diphtheria toxin-like ARTs (ARTDs), consists of seventeen members in humans, of which PARP1-3 are directly involved in the DNA damage response (DDR)(Schreiber et al., 2002; Boehler et al., 2011; Liu et al., 2017; Lüscher et al., 2021). The latter, also termed DNA repair PARPs, are specifically activated by binding to DNA lesions and subsequently ADP-ribosylate a variety of different targets within the vicinity of the damage site (Langelier et al., 2012; Eustermann et al., 2015; Pascal, 2018). Unlike most other PARPs, PARP1 and 2 can PARylate proteins by elongating pre-existing MARylation sites (Figure 1). (ADP-ribose)polymers come in varying lengths and morphologies, linear or branched, which was shown to have physiological effects including the alteration of gene expression, affecting PAR reader recruitment, and signal persistence (Hatakeyama et al., 1986; Aberle et al., 2020; Rack et al., 2021; Reber and Mangerich, 2021). Amongst the DNA repair PARPs, PARP1 is the earliest and most prolific DNA damage sensor with sub-second recruitment onset in laser micro-irradiations experiments (Haince et al., 2008) and is responsible for up to 90% of DNA damage-induced PAR in cells (D’Amours et al., 1999). Targets of the modification include PARP1 automodification as well as other chromatin and repair associated proteins, such as histones (Chapman et al., 2013; Zhang et al., 2013; Daniels et al., 2014; Pic et al., 2014; Teloni and Altmeyer, 2016; Bonfiglio et al., 2017; Palazzo et al., 2018). The locally generated ADP-ribosylation signal serves as a recruitment scaffold for a variety of PAR-binding factors and supports the assembly of the DNA repair machinery (Teloni and Altmeyer, 2016). Moreover, ADP-ribosylation has regulatory roles in the DDR, including facilitating chromatin reorganization and altering transcription (Wei and Yu, 2016; Polo et al., 2019). In comparison, PARP2, a close homolog of PARP1, is recruited to DNA lesions at a slower rate, potentially due to the absence of the N-terminal zinc finger motifs that facilitate PARP1 damage recognition, but persists longer than PARP1 (Perina et al., 2014; Liu et al., 2017; Chen et al., 2018). While both PARP1 and 2 can establish initial modification and elongate these into polymers, the differences in recruitment dynamics and signal production have been suggested to indicate that PARP1 and 2 play only partly overlapping roles in the establishment of the complex and context-specific “PAR code” (Mortusewicz et al., 2007; Liu et al., 2017; Chen et al., 2018). Indeed, PARP1-derived linear PAR, in addition to DNA damage, can activate PARP2 and stimulate the PARP2-dependent production of branched polymers, which are subsequently recognized by histone chaperone APLF and facilitate effective DNA repair (Chen et al., 2018). How this induction of branching is achieved, how it mechanistically differs from the normal, stochastic PARP1 and 2 branching background, whether the branch frequency of PARP1 can be altered, and whether establishment of specific branching patterns is possible remains, as yet, elusive.

Initially, PARP1-3 have been shown to modify glutamate/aspartate residues (Sharifi et al., 2013; Zhang et al., 2013; Daniels et al., 2015; Gibson et al., 2016). Lysine residues have been also suggested, but many of the suggested sites turned out to be mis-assignments (Crawford et al., 2017). Recently, serine residues have been identified as the most abundant acceptor of ADP-ribosylation, especially in the context of DDR (Leidecker et al., 2016; Bonfiglio et al., 2017; Larsen et al., 2018; Palazzo et al., 2018). It was shown that PARP1 and 2 are required, but not sufficient, for serine-ADP-ribosylation (Ser-ADPr). Histone PARylation Factor 1 (HPF1) (Gibbs-Seymour et al., 2016) forms a non-obligate, transient complex with either PARP1 or 2 (PARP1/2), thus enabling modification of serine residues by extending the catalytic center. Moreover, formation of the complex increases the efficiency of the ADP-ribosylation reaction (Figure 1; Bonfiglio et al., 2017; Prokhorova et al., 2021b). Importantly, Ser-ADPr is specifically removed by a single enzyme, ARH3 (Fontana et al., 2017), in conjunction with PARG that acts on PAR chains (Lin et al., 1997; Slade et al., 2011).

This review focuses on Ser-ADPr as the most prominent protein ADP-ribosylation type of the DDR and explains the details of its synthesis and removal, influence on cellular outcomes of DNA damage and the therapeutic potential of targeting Ser-ADPr signaling.

## Histone PARylation Factor 1 as an Auxiliary Factor of PARP1/2

HPF1 was initially linked to DNA repair PARPs due to the presence of a poly(ADPr)-binding zinc finger (PBZ) domain in the orthologs from insects and molluscs (Ahel et al., 2008). Later, it was shown that human HPF1 interacts specifically with PARP1 and 2, and promotes their efficient modification of histones (Gibbs-Seymour et al., 2016). The recruitment of HPF1 to DNA damage sites depends on direct physical interaction with PARP1 and does not require the prior presence of an ADP-ribosylation signal (Gibbs-Seymour et al., 2016; Suskiewicz et al., 2020; Prokhorova et al., 2021b). Loss of HPF1 greatly increases cellular sensitivity to treatment with DNA alkylating agents, such as methyl methanesulfonate (MMS) and sensitizes cells to PARP inhibition (Gibbs-Seymour et al., 2016). HPF1 was further shown to limit PARP1 hyper-automodification *in vivo* and *in vitro*, instead redirecting its catalytic activity toward histones and other substrates (Gibbs-Seymour et al., 2016). HPF1 not only boosts the ADP-ribosylation activity on histones and other targets (see below), but also is the determining factor in shifting PARP1-specificity from Glu/Asp residues to the generation of Ser-ADPr (Bonfiglio et al., 2017). Proteomic and cell-based analyses further confirmed that HPF1 is essential for the widespread Ser-ADPr following DNA damage with targets including histones, PARP1 and hundreds of other proteins (Bonfiglio et al., 2017; Hendriks et al., 2021).

The interaction of HPF1 with PARP1 is strengthened by DNA and NAD^+^, providing a potential mechanism how HPF1, which is estimated to be twenty-times less abundant than PARP1 (Hein et al., 2015; Gibbs-Seymour et al., 2016), could be preferentially recruited to PARP1 molecules that become activated upon detecting DNA damage (Suskiewicz et al., 2020). PARP enzymes directly involved in DNA repair, PARP1-3, are defined by their helical subdomain (HD), an autoinhibitory domain that rapidly unfolds upon recognition of DNA damage, thereby exposing the NAD^+^ binding site (Dawicki-McKenna et al., 2015). Deleting the HD enhances the HPF1:PARP1/2 interaction both *in vitro* and in cells (Suskiewicz et al., 2020), suggesting that this subdomain inhibits HPF1 binding and its DNA-induced unfolding could explain the enhancement of the interaction by DNA breaks.

Recently, the crystal and cryo-EM structures of HPF1 bound to the PARP2 catalytic domain were solved, providing first insights into the structural basis for the HPF1-mediated serine switch (Bilokapic et al., 2020; Suskiewicz et al., 2020). These data were confirmed by NMR and crystallographic analyses of the HPF1:PARP1 interaction (Suskiewicz et al., 2020; Sun et al., 2021). The HPF1:PARP1/2 interaction was found to critically depend upon a conserved aspartate residue (Asp283) in the C-terminal region of HPF1 that contacts His826 in PARP1 (His381 in PARP2) as well as the highly conserved leucine-tryptophan C-terminal residues of PARP1/2 that lock into a groove on HPF1 (Suskiewicz et al., 2020; Rudolph et al., 2021; Sun et al., 2021; Suskiewicz et al., 2021).

Structural and mutational analysis of the HPF1:PARP2 complex also revealed that the HPF1-mediated amino acid preference switch of PARP1/2 can be explained by the provision of a catalytic glutamate residue by HPF1 (Suskiewicz et al., 2020). PARP1 and PARP2 by themselves contain a single catalytic glutamate residue (Glu988 and Glu545, respectively), which was shown to be critical for PAR chain elongation (Marsischky et al., 1995), but this is not sufficient for Ser-ADPr (Bonfiglio et al., 2017). Interaction of HPF1 and PARP1/2 places Glu284 of HPF1 near the catalytic glutamate of PARP1/2 and the NAD^+^ molecule, allowing the formation of a composite active site that is capable of catalyzing efficient Ser-ADPr (Suskiewicz et al., 2020). Glu284 of HPF1 could act as a general base in this reaction, abstracting a proton from the acceptor serine residue in a substrate (Suskiewicz et al., 2020) analogously to a conserved catalytic aspartate found in protein-serine/threonine/tyrosine kinases (Endicott et al., 2012). The deprotonation step is dispensable when the acceptor is a glutamate or aspartate residue, possibly explaining why ADP-ribosylation of acidic residues does not require HPF1. The HPF1:PARP1/2 complex contains a putative peptide-binding cleft with a strong negative charge provided by HPF1 (Suskiewicz et al., 2020), which was suggested to explain the abundance of Ser-ADPr within lysine-serine (KS) consensus motifs (Leidecker et al., 2016; Bonfiglio et al., 2017).

Interestingly, HPF1 also limits auto-PARylation of PARP1/2, leading to the formation of shorter polymers (Gibbs-Seymour et al., 2016; Suskiewicz et al., 2020). Asp283 of HPF1 was shown to occupy the negative-charge binding pocket, which during the PAR chain elongation reaction recognizes the pyrophosphate group of the acceptor ADPr unit (Suskiewicz et al., 2020). As a result, HPF1 binding to PARPs is mutually exclusive with PAR chain formation. This leads to the idea of distinct PAR chain initiation and elongation steps, catalyzed by HPF1:PARP1/2 or PARP1/2 alone, respectively. Indeed, MARylation of histones primed by the HPF1:PARP1/2 complex can be efficiently extended by PARP1 alone (Figure 1; Prokhorova et al., 2021a).

## Reversal of Serine-ADP-Ribosylation By (ADP-Ribosyl)Hydrolase 3

The consumption of the metabolic cofactor NAD^+^, associated with the formation of extensive linear and branched (ADPr)polymers following DNA damage, exerts a high energetic cost, and hence has to be tightly regulated. This cost is partly offset by the degradation of the polymer into free ADP-ribose by macrodomain- or ARH-type hydrolases and subsequent conversion into ATP by ADPr pyrophosphorylase, thus directly supporting ATP-dependent repair processes (Tanuma, 1989; Oei and Ziegler, 2000; Wright et al., 2016; Rack et al., 2020). In addition, ADPr can feed into nucleotide salvage pathways through the conversion into AMP by Nudix hydrolases (Dölle et al., 2013; Rack et al., 2016). Poly(ADP-ribosyl)glycohydrolase (PARG) is the dominant degrader of linear and branched chains, which hydrolzes the ribose-ribose bonds within PAR chains with high efficiency (Figure 1; Hatakeyama et al., 1986; Alvarez-Gonzalez and Jacobson, 1987; Braun et al., 1994; Rack et al., 2021). ARH3 can also degrade linear chains, albeit with a one-to-two orders of magnitude lower activity than PARG and is incapable of cleaving branched PAR (Figure 1; Oka et al., 2006; Drown et al., 2018; Rack et al., 2021). Consequently, PARG is the dominant force controlling PAR chain degradation in cells (Fontana et al., 2017); however, PARG activity is lowered on PAR chains shorter than four ADPr units (Hatakeyama et al., 1986; Barkauskaite et al., 2013). Moreover, PARG cannot hydrolyze the seryl-ADP-ribosyl bond (Slade et al., 2011; Fontana et al., 2017) and ARH3 is the only known human enzyme that can catalyze this reaction (Figure 1). This suggests that PAR signaling is a multi-step process not only on the level of synthesis (incl. initiation, elongation, and branching), but also on that of reversal (incl. cleavage, branch pruning, and termination). This complexity suggests that ADP-ribosylation signaling acts not only as a generic repair factor recruitment scaffold, but is utilized to fine-tune the DDR in a context specific manner. This is further highlighted for example by the diversity of PAR-substructure readers (Teloni and Altmeyer, 2016) or the influence of polymer composition on its stability (Rack et al., 2021). Furthermore, inactivation of both hydrolases is required to induce uncontrolled PAR accumulation with severely increased chain length and abundance (Prokhorova et al., 2021a).

Phylogenetically and mechanistically, PARG and ARH3 belong to distinct families of hydrolases, the macrodomains and (ADP-ribosyl)hydrolases, respectively (Rack et al., 2020). ARH3 is a compact, mainly α-helical orthogonal bundle with a catalytic binuclear Mg^2+^ center situated within the ligand-binding cleft (Mueller-Dieckmann et al., 2006; Pourfarjam et al., 2018; Rack et al., 2018; Wang et al., 2018). Substrate binding was proposed to be gated by a conformationally flexible region, termed Glu41-flap due to the presence of the catalytic Glu41 residue (Pourfarjam et al., 2018). In the auto-inhibitory closed state, Glu41 interacts with Mg_II_, thus locking the active site and sequestering the catalytic residue (Pourfarjam et al., 2018; Rack et al., 2018; Wang et al., 2018). It was recently shown that substrate binding not only displaces Glu41 from Mg_II_ leading to the opening of the Glu41-flap, but actually positions Glu41 in close proximity to Mg_I_, where it contributes to activation of a water molecule for the nucleophilic attack on the scissile bond, which initiates the catalytic cycle (Rack et al., 2021). Moreover, substrate binding induces changes in the coordination of Mg_II_, which adopts a higher-energy square-pyramidal geometry, thus contributing to substrate activation (Rack et al., 2021). In contrast, the PARG structure is composed of a three-layer α/β/α sandwich with a substrate binding groove along the crest of the domain (Slade et al., 2011; Dunstan et al., 2012; Tucker et al., 2012). The catalytic mechanism involves the induction of a strained substrate binding conformation as well as substrate activation by a catalytic glutamate dyad (Patel et al., 2005; Slade et al., 2011; Lambrecht et al., 2015).

Deficiency of PARG and ARH3 leads to sensitivity to DNA damage (Cortes et al., 2004; Mashimo et al., 2013; Shirai et al., 2013). PARG was found to be an essential gene, with deletion leading to embryonic lethality in both mice and flies (Hanai et al., 2004; Koh et al., 2004). Continued culture at 29°C upon pupation allowed a minority (<25%) of flies to survive into adulthood, although these flies showed a progressive neurodegenerative phenotype linked to PAR accumulation in neurons (Hanai et al., 2004). In mice, knock-out of PARG_110_, the longest and primary nuclear isoform, induces a hypersensitivity to exogenous DNA damage (Cortes et al., 2004).

Loss of cellular ARH3 activity, recently described in patients with the autosomal recessive disorder stress-induced childhood-onset neurodegeneration with variable ataxia and seizures (CONDSIAS), was linked with episodic infection-/stress-associated neurological deterioration resulting in impaired or declining cognitive development, physical impairments including muscle weakness, seizures and gait ataxia, and in several cases childhood lethality (Danhauser et al., 2018; Ghosh et al., 2018). ARH3 localizes to the cytoplasm, nucleus, and mitochondria (Oka et al., 2006; Niere et al., 2008), but it has been suggested that its nuclear function is critical to prevent neurodegeneration (Beijer et al., 2021). While the precise molecular causes are not fully understood, accumulation of both chromatin-linked and free PAR was observed (Danhauser et al., 2018; Ghosh et al., 2018; Mashimo et al., 2019) and both processes are linked to aberrant cellular functions. First, cytoplasmic ARH3 protects cells from oxidative-stress induced cell death (parthanatos) by preventing PAR-induced AIF release from the mitochondria (Mashimo et al., 2013). ARH3 thus counteracts PARG by degrading PARG-generated free PAR chains induced by severe oxidative DNA damage (Mashimo et al., 2013), providing a potential therapeutic target not only for CONDSIAS patients, but also other forms of parthanatos-induced cell death, for instance in ischemic brain injury and other neurodegenerative illnesses (Mashimo et al., 2013, 2019). Second, histone ADP-ribosylation was shown to affect other modifications, including acetylation and phosphorylation, and to influence the local histone code (Bartlett et al., 2018; Palazzo et al., 2018; Hanzlikova et al., 2020). Recent cell biological data further suggest that persistent chromatin serine ADP-ribosylation can lead to dysregulated transcription and abnormal telomere structure (Prokhorova et al., 2021a).

## Discussion

While the discovery of Ser-ADPr has greatly expanded the research in the DNA-damage dependent ADP-ribosylation signaling field, our understanding of the exact role of this PTM is still in its infancy. One emerging role of Ser-ADPr is the control of the chromatin state, which is supported by initial findings of cross-talk between histone Ser-ADPr and other canonical histone marks (Bartlett et al., 2018; Prokhorova et al., 2021a). One example stems from histone H3, where neighboring Ser-ADPr and acetylation marks were found to be mutually exclusive (Bartlett et al., 2018; Liszczak et al., 2018). In addition, HPF1 was recently also implicated in regulation of replication. HPF1-directed PARP1 activity was shown to be required for recruitment of XRCC1/DNA ligase 3 complexes, which provide a back-up mechanism for Okazaki fragment ligation, and thus promoting repair of replication-associated DNA damage (Kumamoto et al., 2021). HPF1 also cooperates with the methyltransferase CARM1 to stimulate PARP1 activity and thereby promotes slowing down of replication fork progression (Genois et al., 2021).

So far, the only consequence of site-specific Ser-ADPr that is understood is the effect of the PARP inhibitor response through PARP1 automodification (Prokhorova et al., 2021b). Mutation of PARP1 Ser499, Ser507 and Ser519, or loss of HPF1, leads to greater sensitivity to PARP inhibitors by resulting in increased PARP trapping on chromatin (Prokhorova et al., 2021b). As such, HPF1 loss could be considered a potential biomarker for cancer therapy.

Similarly, ARH3 also emerges as a potential cancer biomarker and drug target, partially due to being the “opposing force” to HPF1. Specifically, either HPF1 deficiency or ARH3 overexpression led to PARP inhibitor sensitivity (Prokhorova et al., 2021b). In line with this, ARH3-deficient cells are sensitive to PARG inhibitors and resistant to PARP inhibitors (Prokhorova et al., 2021b). ARH3 deficiency is therefore a potential novel PARP1 inhibitor resistance mechanism, similar to what has been described for loss of PARG, which causes PARP inhibitor resistance in cancer cells due to stabilization of the PARylation signal (Gogola et al., 2018). Moreover, pharmacological inhibition of ARH3 appears to negatively impact DNA damage repair (Liu et al., 2020). With several lines of evidence pointing at a protective role of ARH3 against neurodegeneration there exists a further pathway to therapeutic application of ARH3 antagonists that can be explored in the future (Danhauser et al., 2018; Ghosh et al., 2018; Mashimo et al., 2019). Deepening our understanding of the opposing forces of HPF1 and ARH3 in the making and breaking of Ser-ADPr will certainly aid our progress in many therapeutically relevant avenues in the future.

## Author Contributions

KS, JGMR, and IA wrote the review. All authors contributed to the article and approved the submitted version.

## Conflict of Interest

The authors declare that the research was conducted in the absence of any commercial or financial relationships that could be construed as a potential conflict of interest.

## Publisher’s Note

All claims expressed in this article are solely those of the authors and do not necessarily represent those of their affiliated organizations, or those of the publisher, the editors and the reviewers. Any product that may be evaluated in this article, or claim that may be made by its manufacturer, is not guaranteed or endorsed by the publisher.
